# Supplementary material for: Individual long non-coding RNAs have no overt functions in zebrafish embryogenesis, viability and fertility
Source: eLife. 2019 Jan 8;8:e40815. doi: 10.7554/eLife.40815 (PMC6347452; doi:10.7554/eLife.40815)
Supplement: Transparent reporting form [file elife-40815-transrepform.pdf]

eLIFE
1st Floor
24 Hills Road
Cambridge CB2 1JP, UK
P 01223 855340
W elifesciences.org
T @elife

eLife's transparent reporting form

We encourage authors to provide detailed information *within their submission* to facilitate the interpretation and replication of experiments. Authors can upload supporting documentation to indicate the use of appropriate reporting guidelines for health-related research (see EQUATOR Network), life science research (see the BioSharing Information Resource), or the ARRIVE guidelines for reporting work involving animal research. Where applicable, authors should refer to any relevant reporting standards documents in this form.

If you have any questions, please consult our Journal Policies and/or contact us: editorial@elifesciences.org.

Sample-size estimation

- You should state whether an appropriate sample size was computed when the study was being designed
- You should state the statistical method of sample size computation and any required assumptions
- If no explicit power analysis was used, you should describe how you decided what sample (replicate) size (number) to use

Please outline where this information can be found within the submission (e.g., sections or figure legends), or explain why this information doesn't apply to your submission:

- No method was used to define the number of lncRNAs to be investigated in this manuscript.

- in-situ RNA hybridizations: No statistical method was used to define the number of embryos to be imaged. Either wild type or homozygous mutant embryos were used, and uniformity of expression pattern was assumed for each genotype.

Replicates

- You should report how often each experiment was performed
- You should include a definition of biological versus technical replication
- The data obtained should be provided and sufficient information should be provided to indicate the number of independent biological and/or technical replicates
- If you encountered any outliers, you should describe how these were handled
- Criteria for exclusion/inclusion of data should be clearly stated
- High-throughput sequence data should be uploaded before submission, with a private link for reviewers provided (these are available from both GEO and ArrayExpress)

Please outline where this information can be found within the submission (e.g., sections or figure legends), or explain why this information doesn't apply to your submission:

eLife Sciences Publications, Ltd is a limited liability non-profit non-stock corporation incorporated in the State of Delaware, USA, with company number 5030732, and is registered in the UK with company number FC030576 and branch number BR015634 at the address 1st Floor, 24 Hills Road, Cambridge CB2 1JP | August 2014

RNA in-situ hybridizations for each transcript was performed once and stated number of embryos were observed.

For each lncRNA the percent reduction in the transcript level relative to control embryos was calculated by (1 - $2^{- \text{Ave. (dd ct)}}$) *100. Three biological replicates and three technical replicates for each lncRNA was used to calculate percent reduction in lncRNA level.
On average for all the investigated lncRNAs 94 ± 6% reduction in the level was observed (Mean ± Standard deviation of all individual values for each lncRNA).

In Figure 2C, Moropholino injection experiment was performed twice. For each morpholino, at least between 50-70 embryo were injected in each round. Images are representative of each group.

In Figure 4E, total of 360 embryos from 4 different couple were used in the analysis. Graph shows mean and SD for four independent measurements. Genotype of fish used in this experiment were confirmed post analysis by PCR.

In Figure S6, the RNA over expression experiment was performed twice. The counts and images are from the second repetition of the experiment. Genotype of adult fish used in this experiment were confirmed by PCR before injection. Embryos form more than two couple per genotype were pooled together and injected. Eggs form the same parents were used for injection of all different mRNAs in this experiment.
Stated number of embryos were scored for phenotype.

eLIFE
1st Floor
24 Hills Road
Cambridge CB2 1JP, UK
P 01223 855340
W elifesciences.org
T @elife

Statistical reporting

- Statistical analysis methods should be described and justified
- Raw data should be presented in figures whenever informative to do so (typically when N per group is less than 10)
- For each experiment, you should identify the statistical tests used, exact values of N, definitions of center, methods of multiple test correction, and dispersion and precision measures (e.g., mean, median, SD, SEM, confidence intervals; and, for the major substantive results, a measure of effect size (e.g., Pearson's r, Cohen's d)
- Report exact p-values wherever possible alongside the summary statistics and 95% confidence intervals. These should be reported for all key questions and not only when the p-value is less than 0.05.

Please outline where this information can be found within the submission (e.g., sections or figure legends), or explain why this information doesn't apply to your submission:

We describe the phenotype of mutants relative to wild-type animals and in this manuscript, no statistical analysis is performed. However the number of analyzed/scored/imaged embryos are indicated throughout the manuscript where needed.

(For large datasets, or papers with a very large number of statistical tests, you may upload a single table file with tests, Ns, etc., with reference to sections in the manuscript.)

Group allocation

- Indicate how samples were allocated into experimental groups (in the case of clinical studies, please specify allocation to treatment method); if randomization was used, please also state if restricted randomization was applied
- Indicate if masking was used during group allocation, data collection and/or data analysis

Please outline where this information can be found within the submission (e.g., sections or figure legends), or explain why this information doesn't apply to your submission:

Experimenter was not blind to the identity of samples collected or measured for any of the experiments shown in this manuscript.

Additional data files ("source data")

- We encourage you to upload relevant additional data files, such as numerical data that are represented as a graph in a figure, or as a summary table
- Where provided, these should be in the most useful format, and they can be uploaded as "Source data" files linked to a main figure or table
- Include model definition files including the full list of parameters used
- Include code used for data analysis (e.g., R, MatLab)
- Avoid stating that data files are "available upon request"

Please indicate the figures or tables for which source data files have been provided:

eLife Sciences Publications, Ltd is a limited liability non-profit non-stock corporation incorporated in the State of Delaware, USA, with company number 5030732, and is registered in the UK with company number FC030576 and branch number BR015634 at the address 1st Floor, 24 Hills Road, Cambridge CB2 1JP | August 2014
